# Spatiotemporal dynamics in the human brain during rest:a virtual brain study

**DOI:** 10.1186/1471-2202-14-S1-P195

**Published:** 2013-07-08

**Authors:** Andreas Spiegler, Enrique Hansen, Viktor K Jirsa

**Affiliations:** 1 Institut de Neurosciences des Systèmes - Inserm UMR 1106 - Aix-Marseille Université, France

## 

Over the past years the ongoing human brain activity at rest came into focus, emphasizing the role of the rest-state activity for brain functions such as planning and perception in both healthy and diseased brains. For instance, it has been shown that the alpha rhythm during rest can be affected (e.g., resetting, entrainment) using stimulations such as sensory or Transcranial Magnetic Stimulations (TMS). However, the origin and the mechanisms underlying the rest-state activity are not yet well understood.

In this study, we focus on the propagation of large-scale brain responses to stimulations such as TMS to identify sub-networks involved in the rest-state.

Using The Virtual Brain [1] we model the dynamics of the human cortex as a network of 16,384 neural masses (NMs), each representing nearly 16 mm^2 ^of the cortical surface. A sub-threshold Hopf oscillator with a Van der Pol term describes the temporal behavior of each NM and a Gaussian kernel defines the spatial interactions among the NMs. We also consider the connections through the white matter extracted from a combination of diffusion spectrum MRI tractography and the CoCoMac database. We systematically stimulate different brain areas and analyze the spatiotemporal responses of the model, using Principal Component Analysis.

**Figure 1:**
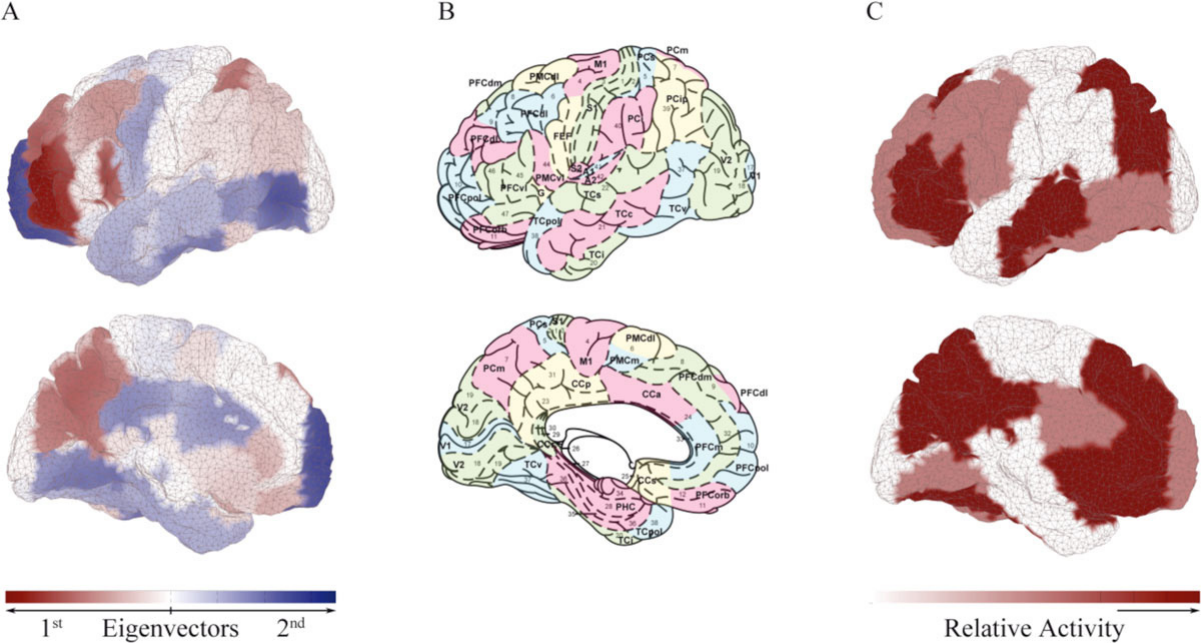
**Network comparison between A. model and C. experimental findings **[3]**using B. Brodmann's parcellation**.

The results provide evidence for the existence of a low dimensional set of networks during rest. Stimulations of brain areas involved in resting-state networks produce stronger and longer lasting responses than stimulations of other areas. We found overlapping of the networks with the dominant connectivity structures as well as with experimentally known resting-state networks (see Figure 1). Our results indicate that resting state networks are critical (closer to the destabilization boundary) which is consistent with experimental studies and recent hypotheses upon the mechanisms generating resting state activity [2].
